# Designing an Equitable Strategy for Allocating Antiretroviral Treatments

**DOI:** 10.1371/journal.pmed.0020069

**Published:** 2005-02-22

**Authors:** Alexander M Capron, Andreas Reis

## Abstract

A study by Wilson and Blower in the February issue of *PLoS Medicine* addressed the issue of ensuring equity in distributing AIDS medications. Reis and Capron discuss the study's implications

## Background

Of the roughly 40 million people living with HIV [1], an estimated 6 million in developing countries urgently need life-saving antiretroviral therapy (ART) [2]. Yet when the 3 by 5 Initiative was launched by the World Health Organization (WHO) and the Joint United Nations Programme on HIV/AIDS (UNAIDS) in December 2003—an initiative that aims to treat 3 million people with HIV in developing countries with ART by the end of 2005—most people with HIV in these countries did not even know their HIV status, and less than 8% were receiving ART. Moreover, even the success of the initiative would still mean that fewer than half of the people who could benefit from such treatment would be receiving it.

Given this gap between what can be done and what needs to be done, the people who set policies and administer programs to provide ART in high-burden countries are faced with difficult questions of distributive justice. Decisions regarding the pricing of ARTs and other care for patients with HIV/AIDS, the distribution of treatment centers, and potential measures to overcome barriers for vulnerable populations will determine who will get access to treatment and who will die. In order to deal with these crucial issues, decision-makers need guidance on how to design policies on equitable access to ART that respect human rights norms and ethical standards.

## Calculating Equitable Access: A New Study

A new study by Wilson and Blower, published in the February issue of *PLoS Medicine* [3], addresses an important dimension of equity in AIDS treatment, namely, the accessibility of health facilities to persons in need. Most published measures of spatial accessibility to health care can be classified into four categories based on the measure of accessibility they use: provider-to-population ratios, distance to nearest provider, average distance to a set of providers, and gravitational (which shows the potential interaction between any population point and all service points within a reasonable distance) [4]. Wilson and Blower developed a mathematical model of the last type that could inform policy-makers' decisions regarding the optimal distribution of treatment sites to ensure equal access by all individuals infected with HIV. Applying this tool to the South-African province of KwaZulu–Natal, Wilson and Blower were able to confirm mathematically the intuitive assumption that using a maximum number of centers, at the least possible distance from most affected populations, would lead to the greatest fairness in the geographical distribution of ART.

## Strengths and Weaknesses of the Study

While the authors suggest that their method could be adapted to take other objectives into account, here they have taken an exclusively egalitarian approach to equity. Although this notion of equity is broadly accepted, other important approaches could have been taken into consideration. Simple equality in access can actually produce inequities (because a fair approach would differentiate among groups in the population according to their different needs); further, under some theories, those who are least advantaged generally should receive a disproportionate share of newly distributed benefits (the maximin principle) [5]. In geographic terms, this goal could be reached by setting up treatment sites preferentially in neglected rural areas or urban slums. Conversely, utilitarian ethics would favor locating treatment sites so as to maximize overall benefits to the population, such as by concentrating treatment in already existing sites that could scale up treatment volume at the lowest cost per patient.

In determining equitable spatial accessibility for the application of their model to KwaZulu–Natal, the authors used a rather rough estimation of HIV prevalence (13% in urban areas and 9% in rural areas). As prevalence greatly varies between specific communities, future studies would certainly benefit from using more disaggregated data where available (see, for example, [6]). Similarly, as the authors recognize, the geographic accessibility of treatment not only is a function of distance, but may be strongly influenced by other factors, such as available transportation options. The concept of “catchment regions” is a valuable one, though still a factor of great uncertainty. Further research is needed to examine the ability and willingness of patients with HIV to travel, taking into account factors such as disease stage, travel times and transportation prices, and socioeconomic factors.

Place matters, but spatial accessibility is only one factor to be overcome in ensuring equitable access to health services. Studies show that even when services are available at a near distance, factors such as temporal accessibility, disease perception, stigmatization, and outright discrimination heavily influence “effective demand” [7]. Moreover, several studies have shown that the price of ARTs may be one of the greatest barriers to access and adherence [8], as even small fees at point of service can prove prohibitive for many people.

## The Future

Wilson and Blower have developed a mathematical model to determine the fair geographical distribution of ART treatment sites and have applied it to the specific setting of KwaZulu–Natal. Despite some methodological and data limitations, such studies can inform policy-makers' decisions regarding the location of HIV services. Since distance to a treatment center is strongly determinant of patients' ability to access care, WHO is developing a service availability mapping tool to monitor relative equity between districts and identify major gaps in service availability, for example, availability of ART and prevention of mother-to-child transmission programs.

Not only is further research needed to refine the spatial accessibility model presented by the authors but careful attention must be paid to other factors that affect access to HIV services and to the underlying assumptions as to what would constitute fair distribution. In a recent guidance document, WHO and UNAIDS recommended that ART programs include special measures to ensure access of vulnerable and marginalized populations and women to ART [9]. The decision-making processes regarding who will get treatment and who won't must be closely monitored for transparency and inclusiveness. Evaluators should also be able to determine the extent to which the scaling-up of HIV/AIDS programs are reaching the target populations and producing equitable results (see Figure 1). To ensure that this process is robust and evenhanded, the guidance document recommends that national AIDS commissions and programs appoint ethics advisory bodies. These ethics committees are to make sure that issues of equity receive attention alongside technical considerations, such as the manner in which ART programs are integrated into the general health system and the identification and training of health personnel, whose absence is often the greatest barrier to adequate HIV care [10].

**Figure 1:**
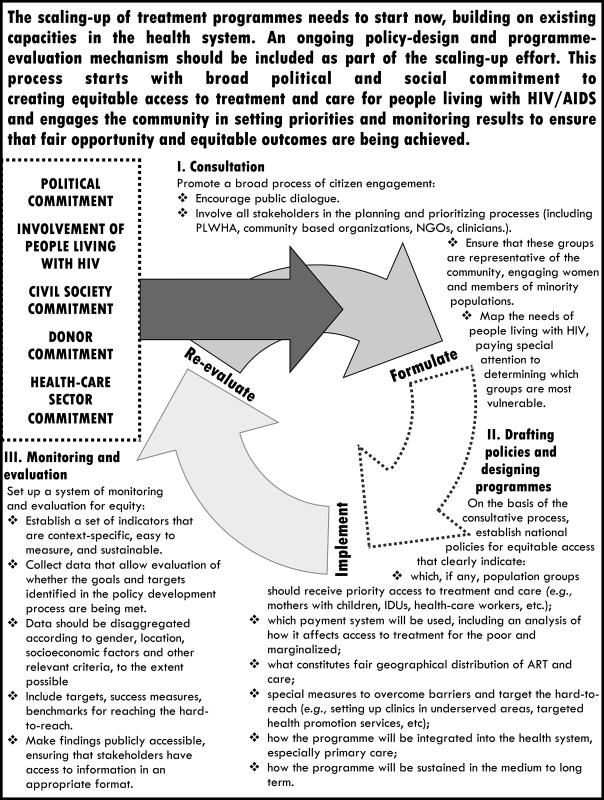
Steps to Equitable Access—The Policy Development Cycle at a Glance IDU, intravenous drug user; NGO, non-governmental organization; PLWHA, people living with HIV/AIDS. (Source: [9])

## Abbreviations

ART: antiretroviral therapy
UNAIDS: Joint United Nations Programme on HIV/AIDS
WHO: World Health Organization
